# Supplementary material for: National trends in utilization and outcomes of elective open and minimally invasive colostomy reversal: A NSQIP analysis
Source: PLoS One. 2025 Jun 25;20(6):e0326963. doi: 10.1371/journal.pone.0326963 (PMC12193832; doi:10.1371/journal.pone.0326963)
Supplement: S1 Table — (DOCX) [file pone.0326963.s001.docx]

|  | Year | Age | Sex | Race | Functional Status | ASA Class | BMI | Ascites | CHF | COPD | Renal Failure | Smoker | Diabetes | HTN | Steroid use | Weight loss |
| --- | --- | --- | --- | --- | --- | --- | --- | --- | --- | --- | --- | --- | --- | --- | --- | --- |
| Overall complications | X | X | X | X | X | X | X | X | X | X | X | X |  |  | X | X |
| Cardiac | X | X | X | X | X | X | X | X | X | X | X | X |  |  | X | X |
| Respiratory | X | X | X | X |  | X |  |  |  | X | X | X | X | X | X | X |
| Infectious |  | X | X | X | X | X | X | X | X |  |  | X | X |  | X | X |
| Wound | X | X | X |  | X | X | X |  |  | X | X | X | X | X |  | X |
| Renal |  | X | X | X | X | X | X |  | X |  |  |  | X | X | X | X |
| Thromboembolic |  | X |  |  | X | X | X |  |  | X |  |  |  |  |  |  |
| Transfusion |  | X | X |  | X | X | X | X | X |  | X | X | X | X | X | X |
| Reoperation | X | X | X |  | X | X |  |  |  | X | X | X | X | X | X | X |
| Operative time | X | X | X | X | X | X | X |  |  | X | X |  | X | X | X |  |
| Length of stay | X | X | X | X | X | X | X |  | X | X | X |  | X | X | X | X |
| Nonhome discharge | X | X | X | X | X | X | X |  | X | X | X | X | X |  |  | X |
| 30-day readmission | X | X | X |  | X | X | X |  | X | X | X | X |  | X | X | X |

**Supplementary Table 1.** Variables Selected via Least Absolute Shrinkage and Selection Operator (LASSO) for Multivariable Modeling. *Abbreviations:* ASA, American Society of Anesthesiologists; BMI, Body Mass Index; CHF, Congestive Heart Failure; COPD, Chronic Obstructive Pulmonary Disease; HTN, Hypertension.
